# Medical Department University of Buffalo

**Published:** 1896-01

**Authors:** Matthew D. Mann, Charles Cary, John Parmenter

**Affiliations:** Committee; Committee; Committee


### IN MEMORIAM.

### Mahlon Bainbridge Folwell, M. D.

MEDICAL DEPARTMENT UNIVERSITY OF BUFFALO.

At a meeting of the Faculty of the Buffalo Medical College, held Wednesday evening, December 11th, the following memorial was adopted and ordered spread upon the minutes :

We, the Faculty of the Medical Department of the University of Buffalo, wish to place on record an expression of the great sorrow which we feel for the loss we have sustained in the untimely death of Dr. M Bainbridge Folwell.

Dr. Folwell has been connected with the College for many years,—first as a student, then as Demonstrator of Anatomy, later as Clinical Professor of Dermatology, and finally as Professor of the Diseases of Children. In all of these capacities he has fulfilled the duties of his position with great ability and untiring faithfulness. We feel his loss most deeply, for we know that in him the College has lost not only a good teacher, but a devoted friend.

While we shall miss him in his connection with the College, to a still greater degree shall we feel his loss as a man and a companion. He was "true and just in all his dealings," faithful and courageous in the discharge of his duties—a man who could always be depended upon to do what he thought was right. His professional abilities were of the highest order. and his social characteristics such as to make him a general favorite. We extend to his wife and family our sincerest sympathy in this their terrible affliction, and share with them in their heartfelt regret that one so necessary to his family, so useful in the community and so endeared to his friends by many kindly acts, should be thus cut off in the prime of life.

MATTHEW D. MANN,
CHARLES CARY,
JOHN PARMENTER,
*Committee.*

